# *Pseudomonas aeruginosa* Consumption of Airway Metabolites Promotes Lung Infection

**DOI:** 10.3390/pathogens10080957

**Published:** 2021-07-29

**Authors:** Sebastián A. Riquelme, Alice Prince

**Affiliations:** Department of Pediatrics, College of Physicians and Surgeons, Columbia University, New York, NY 10032, USA; sr3302@cumc.columbia.edu

**Keywords:** *Pseudomonas aeruginosa*, immunometabolism, succinate, itaconate, PTEN, CFTR, cystic fibrosis, inflammation

## Abstract

Prevailing dogma indicates that the lung of cystic fibrosis (CF) individuals is infected by multiple pathogens due to the abundant accumulation of mucus, which traps most of inhaled organisms. However, this hypothesis does not explain how specific opportunists, like *Pseudomonas aeruginosa*, are selected in the CF lung to cause chronic disease. This strongly suggests that other factors than mucus are accrued in the human airway and might predispose to bacterial disease, especially by *P. aeruginosa*. In this review we discuss the role of macrophage metabolites, like succinate and itaconate, in *P. aeruginosa* pneumonia. We analyze how dysfunction of the CF transmembrane conductance regulator (CFTR) favors release of these metabolites into the infected airway, and how *P. aeruginosa* exploits these elements to induce transcriptomic and metabolic changes that increase its capacity to cause intractable disease. We describe the host and pathogen pathways associated with succinate and itaconate catabolism, mechanisms of bacterial adaptation to these determinants, and suggest how both experimental settings and future therapies should consider macrophage metabolites abundance to better study *P. aeruginosa* pathogenesis.

## 1. Introduction

*Pseudomonas aeruginosa* predominates as a major cause of lung infection and pulmonary pathology in patients with cystic fibrosis [1,2]. While other Gram-negative, often antibiotic resistant organisms, also infect these patients, primarily late in the course of lung disease, *P. aeruginosa* can occur at any stage in cystic fibrosis (CF), but most typically superinfects, and then replaces *Staphylococcus aureus* as the major airway pathogen [3,4]. Impaired mucociliary clearance and dehydrated airway surface fluid is likely to impact overall bacterial clearance in the CF transmembrane conductance regulator (CFTR)-mutant lung [5], but in itself does not explain why *P. aeruginosa*, but rarely *Klebsiellae*, *Escherichia coli, Proteus* or the other common opportunists, is so specific for CF. Metabolomic data derived from human and murine airways suggest that specific airway metabolites and especially reactive oxygen species (ROS) in general, drive the selection of the specific *P. aeruginosa* phenotypes that are associated with intractable infection [6]. 

Opportunistic pathogens, as the name implies, take advantage of local conditions and can adjust gene expression accordingly. This occurs through genetic adaptation, the up or down regulation of specific pathways based upon the environment [6,7]. Bacterial communities respond to secreted molecules involved in transcriptional activation, often through quorum sensing [8]. There is also in vivo selection of mutants, through single nucleotide polymorphisms (SNPs) or the uptake of foreign genes that have favorable characteristics and provide a competitive advantage [9,10]. By studying gene expression in clinical isolates of *P. aeruginosa* from CF patients with established infection, it is possible to follow the in vivo evolution of specific bacterial genes that are important in chronic infection. 

## 2. The Generation of Succinate in the Airway Provides a Preferred Substrate for *P. aeruginosa* Proliferation

**Figure 1:**
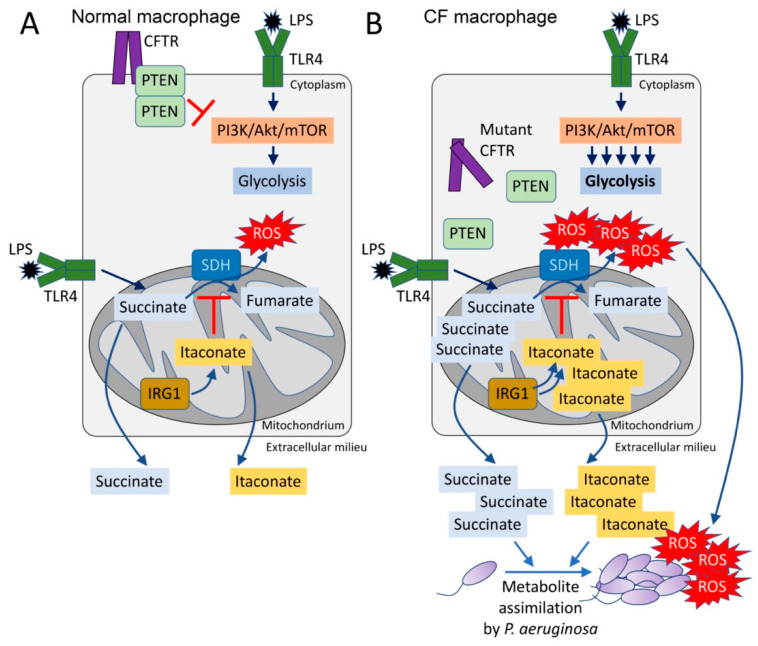
The CFTR-PTEN complex regulates macrophage metabolism and release of immunometabolites. (**A**) During LPS recognition by TLR4, macrophages activate PI3K-Akt-mTOR signaling and glycolysis. The CFTR-PTEN complex regulates this process by inhibiting PI3K function. In parallel, TLR4-LPS interaction promotes anerplerosis, which replenishes the host mitochondria with succinate. Succinate is oxidized by succinate dehydrogenase (SDH) to fumarate, which produces bactericidal ROS. Pro-oxidant SDH activity is regulated by IRG1, which produces itaconate that competes with succinate for the active site of SDH. (**B**) In the absence of the CFTR-PTEN complex, glycolysis is overactivated, as well as succinate oxidation. Itaconate is overproduced to compensate for succinate oxidation, leading to both succinate and itaconate accumulation in the mitochondria. Both metabolites are abundantly released out of the cell, where they can be assimilated by *Pseudomonas aeruginosa*. These organisms also sense ROS, which promotes adaptive changes like biofilm formation.

Historically, bacteria have been classified by their metabolic activity which roughly correlates with the tissues that are major sites of infection [11]. Directed by small RNAs and the *crc* locus, *P. aeruginosa* preferentially consumes succinate over other carbon sources until it is depleted, through a process named catabolite repression [12,13,14,15,16]. Thus, in a setting replete with succinate, such as the inflamed airways, *P. aeruginosa* would have a supply of a major carbon source. Of note, succinate is one of the major metabolites released by macrophages activated by lipopolysaccharide (LPS) [17,18]. Activated macrophages undergo metabolic reprogramming, switching to generate ATP by aerobic glycolysis instead of oxidative phosphorylation (OXPHOS) pathways in the mitochondrion [19]. This metabolic switch repurposes succinate oxidation in the mitochondria not to produce ATP, but, instead, to release bactericidal ROS by action of succinate dehydrogenase (SDH) (complex II) and isocitrate dehydrogenase (IDH, complex I) [19] (Figure 1A). Succinate influx into the active site of SDH is potentiated by anerplerosis, a biochemical process that favors both synthesis and accumulation of succinate from foreign metabolites, such as environmental glutamine [18,19]. Thus, by activating an inflammatory response through LPS, *P. aeruginosa* provides itself with a favored substrate, succinate (Figure 1A). Even in the normal lung, activated macrophages produce and oxidize succinate, which stimulates both stabilization of the hypoxia-induced factor 1α (HIF-1α) and generation of the potent proinflammatory cytokine IL-1β [17,18,19,20]. As described below, a greater production of succinate in cells with CFTR dysfunction may favor *P. aeruginosa* proliferation [21] (Figure 1B). Thus, the CFTR dysfunction and excess proinflammatory signaling fuels *P. aeruginosa* growth, possibly to a greater extent than the recruited immune cells can clear the organisms. 

## 3. Excess Succinate Release Is a Consequence of CFTR-PTEN Complex Dysfunction 

Increased succinate release is a property of CF cells, even in the absence of infection [21]. Cellular metabolic activity is controlled by PI3K and its phosphatase PTEN, an interaction which regulates phosphoinositide abundance, downstream Akt/mTOR signaling and ultimately TCA cycle function in the mitochondrion [22,23,24,25,26]. Functional PTEN is associated with CFTR at the cell membrane, enabling its dimerization and de-phosphorylation [21] (Figure 1A). In the absence of sufficient membrane bound CFTR, PTEN activity is impaired and its brake on mitochondrial generation of succinate is released [21,25]. Mechanistically, lack of membrane PTEN in CF cells favors increased glycolysis and repurposing of mitochondria to produce ROS instead of ATP (Figure 1B). Accumulation of pro-oxidant species activates a compensatory mechanism led by the synthesis of itaconate (*cis*-itaconate), another TCA cycle intermediate produced by *Irg1* (immunoregulatory gene 1, also known as *Acod1*) that inhibits SDH function [21,27] (Figure 1B). Thus, excessive succinate oxidation is prevented by itaconate, but succinate accumulates and permeates towards extracellular compartments where *P. aeruginosa* both senses and assimilates it as carbon source [13,21,28] (Figure 1B). The release of excess succinate by cells with *Cftr* mutations can be corrected by restoring sufficient amounts of PTEN [21]. This also reduces ROS production by mitochondria. Increasing the delivery of CFTR to the membrane would then provide docking sites for PTEN which would also serve to normalize succinate, a response that might be associated with the highly active CFTR modulator therapy, although this has not been directly examined. 

The poor PTEN-CFTR interaction associated with increased succinate in CF is also involved in the altered NF-κB signaling in the airway. PTEN regulates the immunostimulatory functions of the Akt/mTOR pathway [25]. In the absence of sufficient PTEN, the Toll-like receptor 4 (TLR4) adaptor TIRAP/MAL is decreased along with the immunoregulatory p110δ component of PI3K [25]. This results in increased proinflammatory cytokine production during *P. aeruginosa* pneumonia, and may explain the observation that CF infants and some animal models (e.g., ferret) have elevated proinflammatory cytokines in otherwise seemingly normal airways [29,30,31].

## 4. Succinate and the Production of Reactive Oxygen Species in the CF Airway

Succinate, a major component of the TCA cycle, is produced as a function of both bacterial and host metabolism through metabolic pathways that generate oxidants. The specific metabolic pathways used by both host and pathogen to generate ATP produce ROS to differing amounts. While ROS generated intracellularly by phagocytes is important in bacterial killing [32], specially from phagosomes and by complex I and II in mitochondrial compartments, oxidant species are major byproducts of metabolic activity with potentially detrimental effects for both the host airway and the pathogen. Several clinical studies in CF have correlated markers of oxidants stress, such as isoprostane, with inflammation and decreased pulmonary function [33]. As discussed above, lack of normal CFTR function generates excess ROS in many cell types in the airway, independent of infection [34]. ROS inhibits autophagy in CF cells, inducing aggresome formation which adds to inflammation [34]. When sensed by bacteria, excess ROS causes protein aggregation and evokes a major bacterial anti-oxidant response [35]. CF respiratory pathogens have been noted to have significantly increased anti-oxidant capacity as compared with commensal flora or even other respiratory pathogens [36], which is consistent with selection under the increased oxidant stress found in the CF airway. Of note, numerous clinical studies have attempted to therapeutically decrease airway oxidants in CF with a variety of drugs, but without substantial success [37], potentially due to the presence of already ROS-adapted strains that persist beyond the type of treatment used. 

The presence of succinate in the CF airway provides a milieu that supports *P. aeruginosa* proliferation with a preferred substrate. Bacteria grown under conditions of high succinate, like those found in the CF airway, activated pyroptosis and macrophage death, and generated greater amounts of the potent cytokine IL-1β and more succinate [21]. These succinate-adapted strains were better able to colonize the murine airways. However, the endogenous oxidant stress fueled by succinate and generated by *Pseudomonas* metabolic activity as well provides selective pressure for adaptive changes [21]. In response to high succinate in both LB and artificial sputum media (ASM), *P. aeruginosa* PAO1 diverts glucose metabolism via the glyoxylate shunt and Entner-Doudoroff pathway to produce extracellular polysaccharides (EPSs), like alginate, and biofilm [6,21]. These pathways generate fewer oxidants and biofilm itself acts as an oxidant trap. The capacity of both EPSs and biofilm to support *P. aeruginosa* infection in the CF lung has been reviewed [38].

## 5. *P. aeruginosa* Induces and Assimilates Host Itaconate to Cause Long-Term Disease 

**Figure 2:**
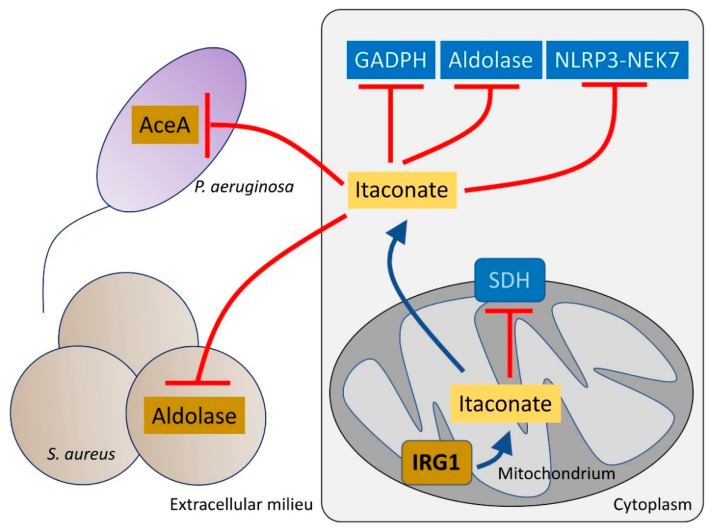
Itaconate controls both host and bacteria metabolism. Itaconate, synthetized by mitochondrial IRG1, inhibits host cell metabolism at different levels. Itaconate can block GADPH, aldolase and the NLRP3-NEK7 complex, which participate in pro-inflammatory signaling. Itaconate also interferes with SDH function, which is required to promote IL-1β synthesis. Once secreted, itaconate blocks the glyoxylate shunt pathway in *P. aeruginosa* by blocking *aceA* activity. In *S. aureus*, itaconate inhibits aldolase, suppressing glycolysis and bacterial proliferation.

The host has numerous pathways to mitigate the generation of oxidants, centering around the transcription factor *Nrf2* and its many downstream targets that promote the anti-oxidant response [39]. One of the metabolites that is released into the airway in response to infection is itaconate, which activates *Nrf2* signaling under LPS stress [40]. Itaconate is a dicarboxylate, structurally similar to both succinate and other TCA cycle determinants and a major metabolite found in the CF airway [41]. In addition to its inhibition of macrophage SDH, itaconate also blocks glycolysis by altering the enzymatic function of both aldolase [42] and glyceraldehyde 3-phosphate dehydrogenase [43] (Figure 2). Itaconate functions as a major immuno-regulatory molecule that resolves inflammation by modulating macrophage metabolism. Itaconate also dampens IL-1β release by blocking NOD-, LRR- and pyrin domain-containing protein 3 (NLRP3) activation [44]. These effects seem to be mediated through NLRP3 decarboxypropylation on cysteine 548 (C548), which is expected to reduce NLRP3 interaction with NEK7, a major inflammasome regulator [44]. 

Itaconate is abundantly produced by macrophages and the host airway after infection with *P. aeruginosa* [21,41]. Itaconate is toxic to many bacterial species, such as *Staphylococcus aureus, Mycobacterium tuberculosis* and *Legionella pneumophila* [45,46,47] targeting the activity of both isocitrate lyase (*aceA*) [48] and aldolase, major metabolic nodes that control the function of the anti-oxidant glyoxylate shunt [49,50] and glycolysis, respectively (Figure 2). However, several important airway pathogens, including *P. aeruginosa, M. tuberculosis* and Aspergillus species can also metabolize itaconate [51]. 

CF-adapted strains of *P. aeruginosa* demonstrated adaptation to itaconate using it as a carbon source, instead of succinate [41]. *P. aeruginosa* harbor three genes devoted to itaconate metabolism: namely, *ict*, *ich* and *ccl.* Expression of these genes is upregulated in response to itaconate, and this loci catabolizes itaconate to produce acetyl-CoA and pyruvate, which fuel OXPHOS function, energy production and generation of biofilm [41,51]. Itaconate is activated for degradation by *ict*, which produces itaconyl-CoA. Then, in a two-steps reaction *ich* first transforms itaconyl-CoA into its isomer mesaconyl-CoA to then hydrate it to form (*S*)-citramalyl-CoA. Finally, *ccl* breaks down (*S*)-citramalyl-CoA to acetyl-CoA and pyruvate, proving the bacteria with pro-energetic intermediates. Clinical isolates from chronic infection, in which itaconate is plentiful, become adapted to both induce and prefer itaconate metabolism, as the clinical strains become impaired in their ability to infect *Irg1*^-/-^ mice [41]. Interestingly, pneumonia caused by a laboratory PAO1 strain, which prefers succinate over itaconate, is independent of host *Irg1* function, illustrating how in vivo adaptation modulates both the immunostimulatory and metabolic preferences of these organisms.

## 6. *P. aeruginosa* Adaptation to Airway Metabolites and the Formation of Biofilm 

One of the most prominent features of *P. aeruginosa* is its ability to form biofilm, and in CF specifically, the selection of mutants that produce copious amounts of the extracellular polysaccharide (EPSs) alginate. While EPSs and the formation of biofilm-encased bacterial communities substantially limit effective phagocytosis and clearance of *P. aeruginosa* from infected airways, metabolic stress itself is a major factor in the selection of variants overproducing EPS, which offers anti-oxidant protection for the bacteria. As an electrophile, itaconate imposes substantial membrane stress on the bacteria [41]. One of the bacterial responses is the selection of variants with diminished surface display of LPS and enhanced production of anti-oxidant EPSs and biofilm. CF isolates behave much in the same way as *lptD* mutants of PAO1 with limited surface display of LPS, decreased immunogenicity, stimulating less TNFα, IL-6 or IL-1β in to the murine airways and increased expression of the genes important in the production of alginate, *algT, algD, algR* and *mucA* [41]. The increased expression of alginate itself is a strong stimulus for host itaconate production, indicating that the adaptive changes to the CF airway do not necessarily result in a loss of immunogenicity, but, instead, a gain of metabolo-stimulatory properties. The capacity of EPSs to stimulate macrophage metabolic reprograming is intriguing, as it discourages the dogma of EPSs being inert molecules that only participate in protecting *P. aeruginosa* from phagocytes. However, it still remains unclear how EPS induces macrophage activation, which must contribute to the ability of many pathogens that abundantly produce surface glycoconjugates to co-opt host metabolic defenses to persist in the human lung. Thus, *P. aeruginosa* and many other opportunists not only activate macrophage metabolic reprograming, succinate release, ROS and inflammation, but also initiates a counter-balancing itaconate-dependent anti-oxidant, anti-inflammatory response in the host that fuel infection instead of its clearance [46,52,53]. 

## 7. *P. aeruginosa* Strains from Chronically Infected CF Subjects Exhibit Adaptation to Host Immunometabolism 

**Figure 3:**
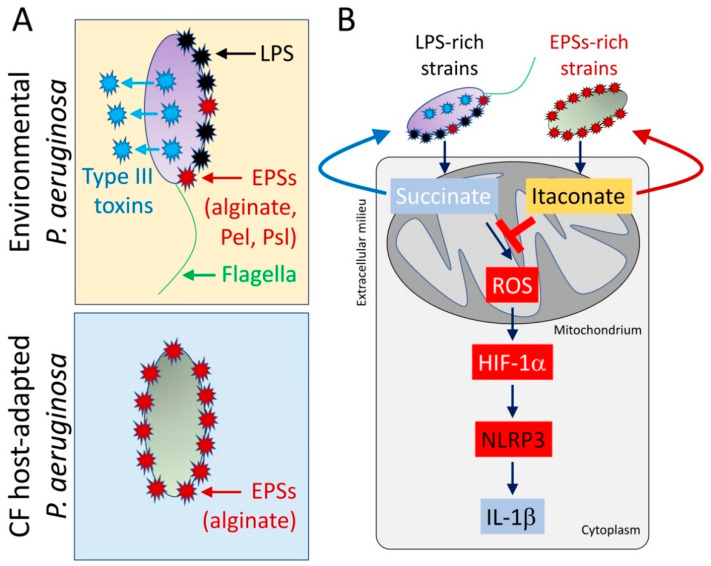
CF host-adapted *P. aeruginosa* isolates evoke itaconate release by airway macrophages. (**A**) In contrast with environmental *P. aeruginosa* strains that produce pro-IL-1β factors like lipopolysaccharides (LPS), type III secretion system’s toxins, and flagella, isolates recovered from the CF airway mostly conserve EPSs production, like alginate. (**B**) CF host-adapted *P. aeruginosa* isolates promote abundant macrophage itaconate signaling and fail to induce the succinate-HIF-1α-NLRP3-IL-1β axis.

Isolates of *P. aeruginosa* from CF chronic infection exhibit many adaptive changes similar to those found in response to growth in macrophage metabolites, like succinate [21]. Some of these clinical strains have SNPs in metabolic genes which enable enhanced growth as well as biofilm formation in the presence of succinate [21]. As has been shown in many phenotypic studies of *P. aeruginosa* strains from CF patients, there are also SNPs in genes affecting flagella, toxins from the type III secretion system, and LPS [9,21,54]. These SNPs either reduce abundance or function of these pathways, compromising their capacity to activate pro-oxidant signaling in the host (Figure 3A). These clinical isolates are less immunostimulatory, and they fail to activate further succinate release, and do not stimulate HIF-1α stabilization or IL-1β expression [21], which is potentiated by their induction of itaconate (Figure 3B). CF adapted strains persist in the murine airway for at least 5 days, in contrast to an equivalent inoculum of the laboratory control strain PAO1 that is lethal in 48 h [21]. Loss of the capacity of these isolates to induce succinate release seems counterintuitive, as succinate is a preferred carbon source for environmental and laboratory strains [12,13,14,15,16]. These data strongly suggest that these organisms progressively adapt to consume other metabolites released by either inflammatory cells or other local microbes, which might derive from pathways required to control excessive airway oxidation and inflammation. Indeed, and as we have discussed above, *P. aeruginosa* isolates from the lung of chronically infected CF subjects exhibit metabolic preferences for itaconate to cause disease, confirming that these organisms adapt to macrophage anti-oxidant routes in order to survive [41]. Interestingly, other major pathogens that co-infect the CF lung with *P. aeruginosa* also produce itaconate, as *Aspergillus* or *Candida* spp. Itaconate is part of their central metabolism, and its synthesis during co-infection might be linked to the ability of *P. aeruginosa* to attach to the human lung. However, this potential mechanism of pathogen-pathogen metabolic interaction requires further research, which, undoubtedly, will enrich our understanding about how different sources of itaconate fuel *P. aeruginosa* disease. Thus, the immunostimulatory as well as the metabolic properties of *P. aeruginosa* change over the course of airway infection, and these modifications might correlate with the type of immune response triggered in the host airway.

## 8. Model Systems in CF and *P. aeruginosa* Pneumonia 

Most of the studies of *P. aeruginosa* pathogenesis in model systems have used laboratory strains, such as PA14, PAO1 and PAK, which are well characterized and fully sequenced. However, pathogenesis studies using clinical isolates illustrate the very substantial differences between these laboratory organisms that reflect properties of environmental strains of *P. aeruginosa* or those taken from the blood stream, versus the organisms that have undergone adaptive changes to long term survival in the human airway [9,21,41,54,55]. Similar discrepancies are observed in the media used to mimic the host airway for in vitro *P. aeruginosa* studies, especially in CF; artificial media containing DNA and mucin reflect many of the conditions expected in the airway but fail to include the major immune cell metabolites, succinate and itaconate or provide ROS that are an inherent component in vivo conditions [56,57,58,59]. The CF airway undoubtedly contains many other immunometabolites and components not yet characterized. Clearly more studies are required to identify how defense cells influence the airway composition before and during bacterial pneumonia. It is also striking that the immune responses evoked by the wild type bacteria, with the activation of HIF-1α and IL-1β and the inflammasome, are not necessarily activated by the more chronic host-adapted strains, indicating that there are many variables that must be considered when using in vitro and in vivo models of infection to study *P. aeruginosa* pathogenesis.

## 9. Targeting *P. aeruginosa* Metabolism as a Therapeutic Strategy 

The tremendous success of *P. aeruginosa* as a human pathogen is in large part due to its large genome, which provides the flexibility that enables the organism to adapt to a wide variety of environmental situations. Metabolic versatility is especially well illustrated in the ability of *P. aeruginosa* to utilize a wide range of potential carbon sources, which, unfortunately, include the very metabolites released by immune cells in their response to infection. Understanding the metabolic properties of this important opportunistic pathogen should help to direct novel therapeutic strategies specific to the properties of the bacteria at the initial or later stages of airway infection. The consumption of succinate in the early infected airway by *P. aeruginosa* should be considered as an important factor when designing therapies to control initial infections, as well as itaconate assimilation during chronic phases of pneumonia. We are close to the realization of a “personalized” approach to bacterial infection. 

Identification of the metabolic activity of the clinical isolates should help to identify the best approach to prevent or treat infection. The highly pro-inflammatory properties of recently acquired environmental *P. aeruginosa,* driven by glycolysis and succinate metabolism should require a distinct immuno-metabolic strategy than would be appropriate for the LPS-deficient, EPS overproducing, itaconate-dependent strains associated with chronic pneumonia. Many immunomodulatory bio-therapeutics are available for other diseases of dysregulated inflammation and might be re-purposed to deal with these infections. Targeting metabolites themselves, while possible, has the added complication of shared metabolic pathways between both host and pathogen. A rational design of drugs including protein structures, specific biochemical pathways like the CFTR-PTEN axis and metabolic targets like the *ict-ich-ccl* locus may provide new approach to anti-bacterial therapy. However, interference with *P. aeruginosa* itaconate assimilation should consider the ability of these organisms to derivatize *cis*-itaconate into *trans*-itaconate (mesaconate), which might compromise the effectiveness of the therapy if this only targets the itaconate isomer produced by the host. Similarly, new therapies using recombinant proteins targeting either host or bacterial metabolism should consider the mechanisms used by *P. aeruginosa* to degrade microbicidal polypeptides, such as elastases from the Type 2 secretion system. Finally, many efflux pumps acquired by these organisms in the host lung might also exclude small molecule drugs aimed to affect *P. aeruginosa* metabolism, suggesting that the new therapies should also consider the interaction between these factors and bacterial transporters. The approach of interfering with both host and bacterial metabolism is attractive in that local tissues like lung, skin, bone and blood, have unique immunometabolic properties that could be exploited to enhance local host defense mechanisms.
